# Manipulating Acoustic Wavefront by Inhomogeneous Impedance and Steerable Extraordinary Reflection

**DOI:** 10.1038/srep02537

**Published:** 2013-08-29

**Authors:** Jiajun Zhao, Baowen Li, Zhining Chen, Cheng-Wei Qiu

**Affiliations:** 1 Department of Electrical and Computer Engineering, National University of Singapore, Singapore 117576, Republic of Singapore; 2 Department of Physics and Centre for Computational Science and Engineering, National University of Singapore, Singapore 117546, Republic of Singapore and; 3 Center for Phononics and Thermal Energy Science, School of Physical Science and Engineering, Tongji University, Shanghai 200092, People's Republic of China

## Abstract

We unveil the connection between the acoustic impedance along a flat surface and the reflected acoustic wavefront, in order to empower a wide wariety of novel applications in acoustic community. Our designed flat surface can generate double reflections: the ordinary reflection and the extraordinary one whose wavefront is manipulated by the proposed impedance-governed generalized Snell's law of reflection (IGSL). IGSL is based on Green's function and integral equation, instead of Fermat's principle for optical wavefront manipulation. Remarkably, via the adjustment of the designed specific acoustic impedance, extraordinary reflection can be steered for unprecedented acoustic wavefront while that ordinary reflection can be surprisingly switched on or off. The realization of the complex discontinuity of the impedance surface has been proposed using Helmholtz resonators.

Refraction in classic optics was recently re-visited from the viewpoints of complex refractive index of a bulky medium^1^, abrupt phase change of an interface^2^, and diffraction theory for gratings^3^. Furthermore, these works shed light on the relation between the reflection and incidence, interpreted as the generalized Snell's law of reflection (GSL)^2^, a novel way to optical wavefront engineering, resulting in promising accomplishments^4^,^5^,^6^,^7^,^8^. In optics, the phase-inhomogeneous metasurfaces realized by thin metallic nanoantennas conserve the wave number along an interface while impose the extra phase accumulation^2^. Fundamental physics is explained by phased antenna array^9^,^10^,^11^.

In principle, GSL is based on Fermat's principle, which holds for all monochromatic waves. However, the luxury of using metallic metasurfaces^2^,^4^ to fulfill the optical phase control is no more available in acoustics due to the limited choice of acoustic materials. Thus, the variable in GSL: phase change on a flat surface becomes an abstract concept in acoustics without any design principle and practical clue. Therefore, it is indispensable and valuable to establish a different principle to manipulate acoustic waves.

In this paper, we establish the framework of acoustic wavefront manipulation by resorting to the specific acoustic impedance (SAI)^12^ inhomogeneity and discontinuity, rather than phase inhomogeneity in terms of wave propagation^1^,^2^. SAI is one of the acoustic properties of a material, more possible to be controllable in reality than that propagation phase. More specifically, we find out the inhomogeneous SAI will generally give rise to one ordinary reflection *p_ro_* and one extraordinary reflection *p_re_*, i.e., *double reflections*. Furthermore, the flat inhomogeneous SAI surface is able to switch on or off *p_ro_* without the influence on its direction, but tweak *p_re_* in the manner of our proposed design principle: impedance-governed generalized Snell's law of reflection (IGSL).

## Results

### Theory: steerable extraordinary reflection and switchable ordinary reflection

**Figure 1:**
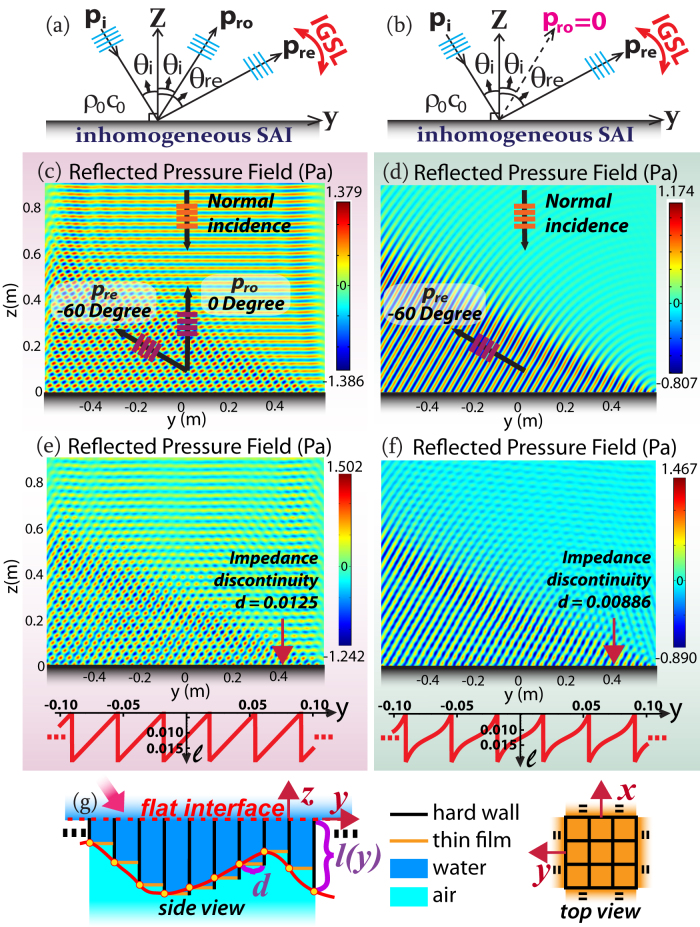
(a) For a flat interface with an inhomogeneous SAI, the angle of *p_ro_*, i.e., *θ_ro_*, is not influenced, while *p_re_* occurs simultaneously and *θ_re_* is controlled by IGSL. (b) If SAI is properly controlled, *p_ro_* is null. (c) Ultrasound with unit amplitude and *ω* = 300 *Krad*/*s* impinges upon SAI surfaces in water. The SAI along the flat surface generates both *p_ro_* and *p_re_* when an arbitrary *A* is chosen in Eq.(1). (d) A particular SAI is chosen according to Eq.(7). 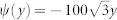 is selected throughout. (e),(f) Simulation results based on impedance discontinuity with relations between *l* and *y* enclosed, corresponding to the cases (c) and (d) respectively. (g) Realization schematics by hard-sidewall tubes of designed lengths.

The inhomogeneous SAI *Z_n_* of the flat surface can be expressed as a complex, whose real and imaginary parts may change spatially. In order to reduce the complexity of modeling as the beginning attempt, we set the real part as a spatial constant. Later we prove that the spatial varying of the real part cannot support our results, which is derived in detail in Supplementary Information. We consider 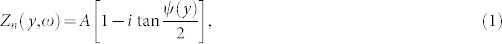
where *A* is an arbitrary constant irrelevant to any spatial change and *ψ*(*y*) is the variable for the imaginary part. Note that *ω*-dependency on the right hand side of Eq. (1) has already been included in *ψ*(*y*). The total acoustic pressure *p* in the upper space satisfies the integral equation: 
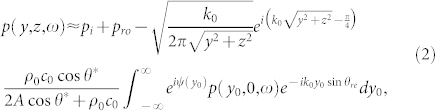
where *p_i_* denotes the incidence; *ρ*_0_ and *c*_0_ are the density and the speed of sound in the upper space in Fig. 1(a); *k*_0_ = *ω*/*c*_0_ is the wave number; θ* is constant; *θ_re_* is the angle of *p_re_*. According to Supplementary Information, both *p_ro_* and *p_re_* exist for a general *A*, implying the unusual *double reflections*: 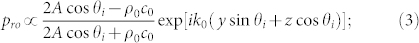

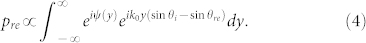


After applying the first-order approximation and the stationary phase approximation to Eq. (4), the relation between *θ_re_* and the incident angle *θ_i_* is unveiled: 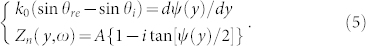
Note that based on our derivation, only when the inhomogeneous SAI along the flat surface is expressed in form of Eq. (1) can our IGSL survive. Although IGSL's appearance is similar to GSL^1^,^2^,^4^, its physical meaning of *ψ*(*y*) are dramatically different. Fundamentally, the variable of our IGSL Eq. (5) is about the value of surface acoustic impedance instead of the abrupt propagating phase change. Moreover, IGSL only serves to steer *p_re_* at will, with no influence on the direction of *p_ro_*, as illustrated in Fig. 1(a). In Supplementary Information we highlight the irrelevance between GSL and our proposed IGSL. In addition, GSL mentions the extra accumulated phases along wave-propagation paths, but it is still relying on graphical methods to find out the relation between the configuration of the passive antenna array and the needed phase in optics^2^. However, we do not have the passive antenna in acoustics. Here, IGSL Eq. (5) and Eq. (1), serving as an explicit design rule, provide us the feasible way based on a different mechanism in acoustics.

Eq. (5) also sheds the light on an extreme angle (similar to critical angle): 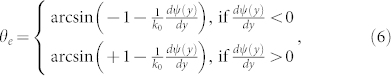
above which *p_re_* becomes evanescent in the upper space. Eq. (6) holds only when 
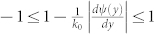
. Otherwise, *p_re_* becomes evanescent.

Usually, both *p_ro_* and *p_re_* will coexist as shown in Fig. 1(a), suggesting *double reflections*, while IGSL only controls *θ_re_*. Hence, it is interesting to eliminate *p_ro_* as shown in Fig. 1(b), by means of a particularly selected value of *A*. Eq. (3) suggests that *A* = (*ρ*_0_*c*_0_)/(2 cos *θ_i_*) can make *p_ro_* vanish, i.e., *p_ro_* is switched off, as shown in Fig. 1(b). The corresponding SAI of the flat surface then becomes 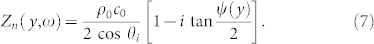


### Verification: continuous impedance and discontinuous impedance

Supposing the gradient of *ψ*(*y*) along the flat interface is constant, we notice Eq. (4) turns out to be a Dirac Delta without approximation. From Eq. (5) we predict the wavefront of *p_re_* will propagate in the form of a plane acoustic wave, independent of *y*. We select water (*ρ*_0_ = 1 *kg*/*m*^3^; *c*_0_ = 1500 *m*/*s*^12^) as the background medium, *ω* = 300 *K rad*/*s* as the circular frequency, 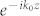
as the normal incident plane ultrasound, and a linear form 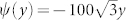 in Eq. (7).

*θ_re_* is theoretically found to be −60° by IGSL, validated by our simulation in Fig. 1(d). *p_ro_* is thoroughly suppressed thanks to the specific *A* chosen according to Eq.(1). In contrast, in Fig. 1(c), the same parameters are kept except for another *A*, whose value is arbitrarily taken to be *ρ*_0_*c*_0_. It clearly shows that *p_ro_* occurs and interferes with *p_re_*, but *p_re_* still keeps the same, verifying our theoretical formulation. In terms of phenomena, the designed inhomogeneous SAI Eq. (1) essentially implies the changes of both the propagating phases and amplitudes, only by which the effect of double reflections may occur. In terms of physics, the extra momentum supplied by the metasurface is employed to compensate the momentum mismatch between the incident acoustic beams and the diffracted beams. Therefore, for the double backward propagating beams, *p_ro_* is the most pervasive specular reflection, while *p_re_* is attributed to the diffraction of higher order.

**Figure 2:**
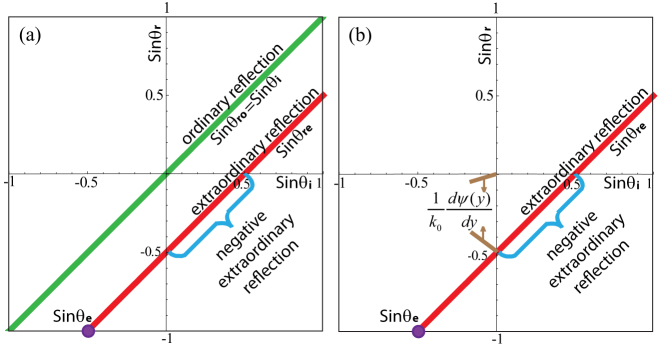
sin*θ_ro_*_, *re*_ versus sin *θ_i_* when *k*_0_ = 10 *rad*/*m* and *ψ*(*y*) = −5 *y*. *p_ro_* and *p_re_* emerge simultaneously in (a). In (b), only *p_re_* occurs for the same parameters of (a) except *A*. The purple dot denotes sin *θ_e_* in Eq. (6).

Fig. 1(d) suggests the possibility of negative reflection for *p_re_*, which is further verified for oblique incidence in Fig. 2. In Fig. 2(a), because of the inhomogeneous SAI and the arbitrary *A* in Eq. (1), both *p_ro_* and *p_re_* occur. Fig. 2(b) depicts the same situation except for *p_ro_* being switched off as a result of the specifically chosen *A* according to Eq. (7), while the red line *p_re_* stays the same as that in Fig. 2(a). The blue braces represent the region of negative *p_re_*. It is noteworthy that *p_re_* does not exist if *θ_i_* is beyond the extreme angle *θ_e_* = −30° in Eq. (6), corresponding to the purple dots. One field simulation is provided in Supplementary Information.

As depicted in Fig. 1(g), we propose one plausible realization schematic for the general SAI of Eq. (1), where all hard-sidewall tubes with one pressure-release termination are gathered and juxtaposed perpendicular to the flat interface. Observed at the top view, each tube has a square cross section whose width is *d*, with four enclosed hard sidewalls (black). Then observed at the side view, the upside open termination of each tube constitutes an effective SAI pixel of the interface, while the other end sealed by a thin film (orange) serves as the pressure-release termination^12^. The upper space and the interior of each tube are filled with water, without separation. The light blue indicates air downside, which is isolated from water by the thin film.

The SAI of each tube at the opening facing the upper space is^12^: 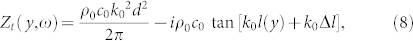
where *l*(*y*) is the length of each tube and 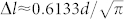 is the effective end correction. By comparison of Eq. (1) and Eq. (8), it is required that *A* = *ρ*_0_*c*_0_*k*_0_^2^*d*^2^/(2*π*) and *A* tan[*ψ*(*y*)/2] = *ρ*_0_*c*_0_ tan [*k*_0_*l*(*y*) + *k*_0_Δ*l*], leading to the value of the spacing *d* for impedance discretization and the dependance between *l*(*y*) and *ψ*(*y*): 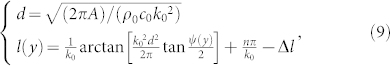
where the arbitrary integer *n* is required to be set suitably to make *l* a positive value. Thus, the change of *ψ* along *y*, representing the control of *p_re_*, is interpreted as the change of *l*, implying one straightforward realization based on discontinuous impedance. Thus, the inhomogeneity of the acoustic impedance is strictly paraphrased into the inhomogeneity of the tube-array structure, resulting in our acoustic metasurface. At the side view in Fig. 1(g), the solid red contour indicates one arbitrary function of *l*(*y*) calculated from Eq. (9). Based on the discretization *d* calculated from Eq. (9) as well, we are able to find *d* and the corresponding height *l*(*y*), marked with the yellow dots. Note that the top of the tube array is aligned into a flat surface (red dashed line), above which acoustic waves impinge. Thus, the change of tube lengths will not affect the flatness of the surface. In addition, thanks to the property of the arc-tangent in Eq. (9), the tube-array metasurface is within a thin layer without the space-coiling-up technique^13^. It is also noteworthy that because of the intrinsic differences between optics and acoustics, so far we cannot obtain the mechanism-analog of the optical metasurface, which is based on resonances and independent with the thickness or effective propagating lengths, but we can achieve the phenomenon-analog in acoustics using the tube array. In principle, because tubes can be regarded as Helmholtz resonators, complex SAI at each pixel can be realized by a suitable arrangement of resonators, as the analog of the complex electric impedance realized by the combination of resistance, capacitance and inductance. In addition, we know that only the real part, the electric resistance, consumes energy while the imaginary part does not. In the same manner in acoustics, the energy loss is theoretically only attributed to the real part of the surface complex SAI in Eq. (8), i.e., the loss in our case is caused by the energy consumption from the tube array.

Using this method, we reproduce Fig. 1(c)(d) by realistic impedance discontinuity, so as to verify our proposed realization. In Fig. 1(e)(f), *d* = 0.0125 and 0.00886 are selected respectively according to Eq. (9), and the corresponding contours of the tube length *l* in terms of the location *y* are illustrated as the red lines, respectively. Fig. 1(e) shows strong interference between *p_re_* and *p_ro_* while Fig. 1(f) shows the nearly undisturbed *p_re_*, coinciding with Fig. 1(c) and (d) respectively.

### Application: acoustic illusion and ipsilateral focusing

**Figure 3:**
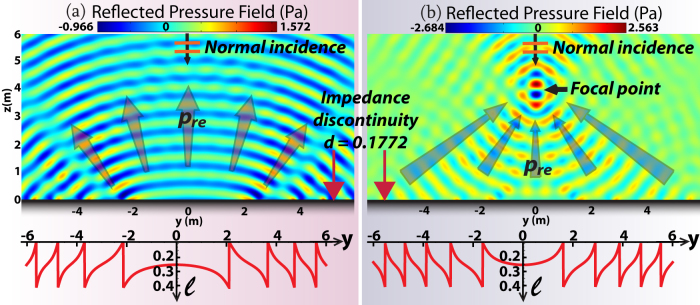
Wavefront metamorphosis via SAI interface, with impedance discontinuity *d* = 0.1772. A plane acoustic wave of *θ* = 15 *Krad*/*s* is normally incident in water. Only reflected acoustic pressure is plotted. (a) The SAI of Eq.(7) with ψ(*y*) = 0.7 *y* ^2^ is set along the flat surface. *p_re_* diverges into a curved wavefront. (b) The SAI of Eq.(7) with 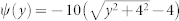 is set. *p_re_* converges to a focal point in the two-dimensional case.

To demonstrate IGSL's capability of designing novel acoustic devices, we metamorphose acoustic pressure fields everywhere through SAI manipulation as simulated in Fig. 3. This deceptive effect is obtained by manipulating plane wavefronts into wavefronts generated by a virtual reflector or focusing illumination, governed by the control of *p_re_*, i.e., IGSL. Under these scenarios, we need to consider nonlinear forms of ψ(*y*). New phenomena are thus expected when *θ_re_* becomes spatially varying.

It is found that the acoustic deception can be created via IGSL, e.g., ψ(*y*) = 0.7 *y*^2^ in Eq. (7), resulting in *p_ro_* = 0. Correspondingly, *θ_re_* in Fig. 3(a) is a position-dependent function sin *θ_re_* = 0.14 *y*, in which case *p_re_* fans out as demonstrated in Fig. 3(a), verifying our theory. Here the spacing *d* for impedance discretization is 0.1772 and the relations between *l* and *y* derived from Eq.(9) are enclosed in Fig. 3. Therefore, IGSL can be employed to camouflage a flat surface as if there were a curvilinear object at the origin instead of the physical planar interface. The dual effect allowing a curved reflector to mimic a flat mirror, by manipulating the convex wavefronts into planar wavefronts, was reported in plasmonic regime^19^.

Furthermore, the SAI can be designed to make acoustic waves reflected by a planar interface focused as well. In optics, a flat lens with metallic nanoantennas of varying sizes and shapes can consequently converge the transmitted light to a focal point^5^,^6^. Note that the optical focusing controlled by optical GSL is on the other side of incoming lights, i.e., on two sides of the flat surface^5^,^6^ in the transmission mode. In acoustics, we employ an inhomogeneous SAI flat surface to focus *p_re_*, in the reflection mode by IGSL without *p_ro_*.

This *ipsilateral focusing* in Fig. 3(b), is thus obtained in the planar geometry in acoustics for the first time. In Eq. (7), a hyperbolic form is set: 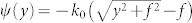 (*f* being the given focal length^20^) for the SAI of the flat interface. *p_re_* from different angles constructively interferes at the ipsilateral focal point, as if the waves emerge from a parabolic surface. The parameters in Fig. 3(b) are the same as those in Fig. 3(a) except for the specific hyperbolic SAI form 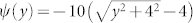, with the designed focal point at (*y* = 0, *z* = 4) and *p_ro_* suppressed. In addition, the simulated acoustic pressure by impedance discretization at the focal point is well confined at (*y* = 0, *z* = 4).

Interestingly, the imaging at the same side was previously presented for electromagnetic waves^14^,^15^. In^14^, it demands strong chiral materials filled in the whole upper space. The same-side imaging is only a partial imaging, i.e., only one circularly polarized wave being imaged and the other being reflected ordinarily. In acoustics, our ipsilateral imaging is achieved by translating all the stringent requirements of the half-space chiral materials into an inhomogeneous impedance surface. In electromagnetism, ipsilateral imaging can be achieved as well by surface gratings^15^ or antenna arrays. However, the polarization of incident electromagnetic waves is always closely related to the effect of focusing. Therefore, the ipsilateral imaging in acoustics by IGSL has no polarization constraints thanks to the acoustic wave nature, i.e., longitudinal vibration.

### Application: conversion from propagating acoustic waves to surface acoustic waves

**Figure 4:**
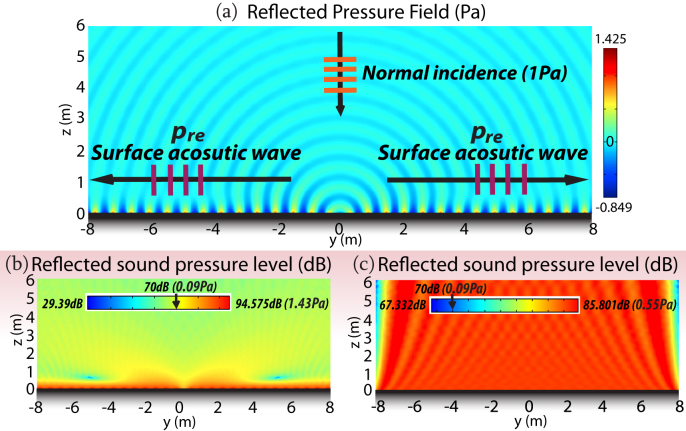
Conversion from PAWs to SAWs via SAI interface. The PAW with unit amplitude and *ω* = 15 *Krad*/*s* is normally incident in water. Only reflected acoustic pressure is plotted. (a) The SAI of Eq.(7) is set to be ψ(*y*) = −11 *y* for *y* < 0 and ψ(*y*) = 11 *y* for *y* > 0. SAWs are bifurcated at the origin and confined near the surface. (b) The reflected sound pressure level of (a). (c) The reflected sound pressure level when a homogeneous SAI is adopted instead.

Beyond the acoustic-field metamorphosis, we further establish a kind of acoustic cognitive deception about a SAI surface converting propagating acoustic waves (PAW) to surface acoustic waves (SAW) in Fig. 4, by means of IGSL. The extreme angle 0° in Eq.(6) demands ψ(*y*) = ± 10 *y*. Therefore, we set the SAI of Eq. (7) slightly over that extreme, e.g., ψ(*y*) = −11 *y* for *y* < 0 and ψ(*y*) = 11 *y* for *y* > 0 are set along the flat interface symmetrically with respect to the *z*. In Fig. 4(a), the bidirectional surface acoustic waves are attributed to the coupling effect governed by the diffracted evanescent *p_re_* which propagates along the metasurface^16^. Owing to the inhomogeneous SAI interface, the ideally perfect conversion comes true in acoustics except for a little diffraction. Physically, the SAI along the flat surface provides an extra momentum to compensate the momentum mismatch between propagating waves and surface waves in acoustics, resulting in the high efficiency conversion. In contrast, if one uses a constant SAI Eq.(7) with ψ(*y*) = 11 along the flat surface (the homogenous SAI does not generate *p_re_*; only *p_ro_* occurs), the reflected sound pressure level in Fig. 4(c) is almost uniformly spread over the space.

Fig. 4(b) clearly demonstrates that the acoustic field is well confined in the region close to the interface and attenuated quickly to around 0 *Pa* away from the interface, revealing the nearly perfect conversion. Interestingly, it shows in^17^ that the electromagnetic-varying metasurface is able to prevent the propagating electromagnetic waves from being reflected back to the upper space. Hence, our PAW-SAW conversion in acoustics, originating from a distinguished mechanism, is differentiated from^17^.

In Fig. 4, we notice such technology is functional as an alternative invisible acoustic cloak by trapping the acoustic field in the vicinity of the coating, resulting in much lower signal of reflection. It may pave the avenue to the large size acoustic invisibility since it is only dependent on the surface technique instead of wave-interaction based metamaterial acoustic cloaking^18^. It will also be promising to consider the time-varying surface technique in acoustics with nonreciprocal diffraction^21^ in the future.

## Discussion

Here, IGSL is established for novel manipulation of acoustic wavefronts. Due to the lack of abrupt-phase-changing surface structures in acoustics, we resort to specific acoustic impedance as the variable to tweak the reflection. IGSL, which can simultaneously generate the switchable *p_ro_* and the steerable *p_re_*, provides us the explicit connection between our designed SAI and the reflected field, serving as the design rule in acoustics. We not only demonstrate intriguing acoustic manipulations but also provide insightful realization schemes. As a few examples, we demonstrate acoustic disguise, acoustic planar lens, acoustic ipsilateral imaging and acoustic PAW-SAW conversion. These novel effects will inspire new technologies on acoustic wave engineering, leading to unprecedented applications.

## Methods

For theoretical derivations, we used Green's function, the integral equation Eq. (2) and Born approximation. The detailed theoretical development is elaborated in Supplementary Information. For the numerical calculations, we used the Finite Element Method by means of COMSOL Multiphysics. The left, right and top sides of the meshed domain are set as plane wave radiation conditions, while the bottom side is set as the impedance boundary with a certain value.

## Author Contributions

J.Z. and C.W.Q. developed the theory, performed the numerical experiments, and prepared the manuscript. B.L. and Z.C. contributed in the analysis. All authors edited the manuscript. C.W.Q. conceived the idea.

## Supplementary Material

Supplementary Information Supplementary information
