# Supplementary material for: Manipulating Acoustic Wavefront by Inhomogeneous Impedance and Steerable Extraordinary Reflection
Source: Sci Rep. 2013 Aug 29;3:2537. doi: 10.1038/srep02537 (PMC3756330; doi:10.1038/srep02537)
Supplement: Supplementary Information [file srep02537-s1.pdf]

Supplementary Information: Manipulating Acoustic Wavefront by Inhomogeneous Impedance and Steerable Extraordinary Reflection

Jiajun Zhao[1,2], Baowen Li[2,3], Zhining Chen[1], and Cheng-Wei Qiu[1,*]

[1] *Department of Electrical and Computer Engineering, National University of Singapore, Singapore 117576, Republic of Singapore*

[2] *Department of Physics and Centre for Computational Science and Engineering, National University of Singapore, Singapore 117546, Republic of Singapore and*

[3] *Center for Phononics and Thermal Energy Science, School of Physical Science and Engineering, Tongji University, Shanghai 200092, People's Republic of China*

(Dated: May 8, 2013)

I. DERIVATION OF IGSL IN ACOUSTICS

We mathematically derive the connection between the interface specific acoustic impedance (SAI) and the manipulation of wavefronts, which gives birth to the proposed impedance-governed generalized Snell's law of reflection (IGSL) as the design rule of SAI. In addition, we mathematically predict the double reflections and the situation when the ordinary reflection can be switched off.

We assume the time-harmonic factor in this appendix is $e^{-i\omega t}$, where ω is the circular frequency, and the coordinate system is that in Fig. 1(a). The incident acoustic pressure can be expressed as:

$$p_i(y,z,\omega) = p_{i0}(\omega)\exp[ik_0(y\sin\theta_i - z\cos\theta_i)], \tag{1}$$

where $k_0 = \omega/c_0$ is the wave number in free space, θ_i the incident angle and $p_{i0}(\omega)$ the amplitude. $Z_n(y,\omega) = p(y,0,\omega)/[\mathbf{n}\cdot\mathbf{v}(y,0,\omega)]$ as the specific acoustic impedance (SAI) [1] of a locally reacting boundary is laid at the interface, where $\mathbf{n}$ is the unit vector opposite to z direction and $\mathbf{v}$ is the acoustic velocity. The boundary condition of this problem can be paraphrased as [2]:

$$\frac{\partial}{\partial z}p(y,0,\omega) + ik_0\beta(y,\omega)p(y,0,\omega) = 0, \tag{2}$$

where $\beta(y,\omega) = \rho_0 c_0/Z_n(y,\omega)$ (ρ_0 and c_0 being the given density and sound speed respectively in the upper space) is the normalized acoustical admittance of the locally reacting surface.

We expand β to be $\beta(y,\omega) = \tilde{\beta}(y,\omega) + \beta_0(\omega)$, where β_0 is a real constant. The ordinary reflection is expressed as:

$$p_{ro}(y,z,\omega) = p_{i0}(\omega)R(\theta_i,\beta_0)\exp[ik_0(y\sin\theta_{ro} + z\cos\theta_{ro})], \tag{3}$$

where R is the reflection coefficient and θ_{ro} the angle of p_{ro}. Because p_{ro} observes the usual Snell's law, $\theta_{ro} = \theta_i$. In order to find the expression of R, we introduce the constant SAI:

$$Z_0(\omega) = \frac{\rho_0 c_0}{\beta_0(\omega)} = \frac{p_i(y,0,\omega) + p_{ro}(y,0,\omega)}{\mathbf{n}\cdot\mathbf{v}_i(y,0,\omega) + \mathbf{n}\cdot\mathbf{v}_{ro}(y,0,\omega)}, \tag{4}$$

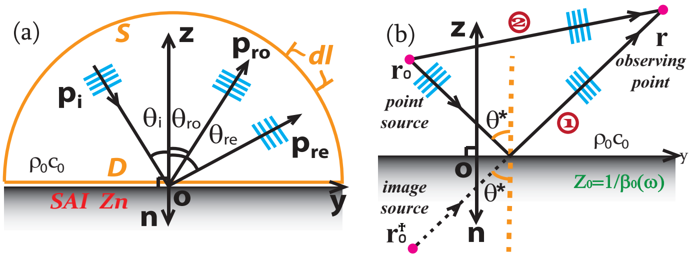

FIG. 1: (a) Illustration for some notations. The orange line indicates the contour of the Green's integral. S is the semicircular contour; D is the flat one along the surface. p_i, p_{ro} and p_{re} denote the incidence, the ordinary reflection, and the extraordinary reflection, respectively. $\mathbf{n}$ is the unit vector opposite to z direction. Z_n is set for the flat interface ($z = 0$). (b) Schematic diagram for the effective paths of acoustic radiation. The introduced θ^* can be interpreted as the effective incident angle. $\mathbf{r}$, $\mathbf{r}_0$, and $\mathbf{r}_0^\dagger$ are the location vectors for the point source, the image source and the observer, respectively.

where $\mathbf{n}$ is the normal vector indicated in Fig. 1(a), $\mathbf{v}_i$ and $\mathbf{v}_{ro}$ the acoustic velocities of p_i and p_{ro}. Substituting Eq.(1) and Eq.(3) into Eq.(4) and applying Euler equation $\rho_0 \frac{\partial}{\partial t}\mathbf{v} = -\nabla p$, we obtain:

$$R(\theta_i, \beta_0) = \frac{\cos\theta_i - \beta_0(\omega)}{\cos\theta_i + \beta_0(\omega)}. \tag{5}$$

In Fig. 1(a), the total acoustic field can be written in the integral form:

$$p(y,z,\omega) = \oint_{S+D} [G(y,z,\omega;y_0,z_0)\frac{\partial}{\partial n_0}p(y_0,z_0,\omega) - p(y_0,z_0,\omega)\frac{\partial}{\partial n_0}G(y,z,\omega;y_0,z_0)]dl, \tag{6}$$

where $dl(y_0, z_0)$ is the infinitesimal segment along the integral contour, $\mathbf{n}_0 = \mathbf{n}(y_0, z_0)$ and $G(y, z, \omega; y_0, z_0)$ is the Green's function corresponding to the following partial differential problem:

$$\nabla^2 G + {k_0}^2 G = -\delta(y-y_0)\delta(z-z_0), z > 0; \left[\frac{\partial}{\partial z_0}G + ik_0\beta_0(\omega)G\right]\Big|_{z_0=0} = 0. \tag{7}$$

When the radius of the semicircular contour S approaches ∞, we can regard the contour integral along S is mainly contributed by p_i and p_{ro}. Therefore Eq.(6) changes into

$$p(y,z,\omega) = p_i(y,z,\omega) + p_{ro}(y,z,\omega) - \int_{-\infty}^{\infty} [G\frac{\partial}{\partial z_0}p(y_0,z_0,\omega) - p(y_0,z_0,\omega)\frac{\partial}{\partial z_0}G]dy_0. \tag{8}$$

We can simplify Eq.(8) by substituting Eq.(7) and Eq.(2) into it. By defining the last part in Eq.(9) as the extraordinary reflection $p_{re}(y, z, \omega)$, which is the unique extra component beyond p_{ro}, we obtain

$$p_{re}(y,z,\omega) = ik_0 \int_{-\infty}^{\infty} \tilde{\beta}(y_0,\omega)p(y_0,0,\omega)G(y,z,\omega;y_0,0)dy_0. \tag{9}$$

The explicit solution of $G(y, z, \omega; y_0, z_0)$ in Eq.(7) is

$$G = \frac{i}{4}H_0^{(1)}(k_0|\mathbf{r}-\mathbf{r}_0|) + \frac{i}{4\pi}\int_{-\infty}^{\infty}\frac{1}{k_z}\frac{k_z - \omega\beta_0/c_0}{k_z + \omega\beta_0/c_0}\exp[ik_z(z+z_0) + ik_y(y-y_0)]dk_y, \tag{10}$$

where $\mathbf{r} = (y, z)$, $\mathbf{r}_0 = (y, z)$, and ${k_0}^2 = {k_y}^2 + {k_z}^2$. When $\mathbf{r}$ is away from the surface D, $k_z \approx k_0 \cos\theta^*$ holds, where θ^* is introduced as a constant. Via this approximation and another definition $\mathbf{r}_0^\dagger = (y_0, -z_0)$, it turns out that [3]

$$\cos\theta^* \approx \frac{z-(-z_0)}{\left|\mathbf{r}-\mathbf{r}_0^\dagger\right|} \approx constant. \tag{11}$$

Through Eq.(11), it can be obtained that

$$\frac{k_z - \omega\beta_0/c_0}{k_z + \omega\beta_0/c_0} \approx \frac{\cos\theta^* - \beta_0(\omega)}{\cos\theta^* + \beta_0(\omega)} \approx constant \approx R(\theta^*, \beta_0). \tag{12}$$

Applying Eq.(12) into Eq.(10) and using the formula of the cylindrical wave expansion in terms of plane waves, we approach a neat form of the Green's function:

$$G(y, z, \omega; y_0, z_0) \approx \frac{i}{4} H_0^{(1)}(k_0 \left|\mathbf{r} - \mathbf{r}_0\right|) + R(\theta^*, \beta_0)\frac{i}{4} H_0^{(1)}(k_0 \left|\mathbf{r} - \mathbf{r}_0^\dagger\right|), \tag{13}$$

where $H_0^{(1)}(\cdot)$ the Hankel function of the first kind[4].

From the physical insight into Eq.(13), the first part of G is the direct contribution of the point source to the observer through path 2 in Fig. 1(b). The second part is the product of the Green's function excited by the image source and the reflection coefficient R, denoting p_{ro}. According to our interpretation, Fig. 1(b) illustrates path 1 and path 2, visualized as p_{ro} and p_{re} respectively[5]. Due to the expression of R, we figure out that θ^* is the effective incident angle regarding to Fig. 1(b). Furthermore, it is reasonable to say that the major contribution of the integral in Eq.(10) is attributed to the vicinity of θ^*, in which way R can be regarded as a constant and put outside the integral.

By far-field approximation, we are able to get these expansions:

$$\mathbf{r} \cdot \mathbf{r}_0 = r(y_0 \sin\theta + z_0 \cos\theta); \mathbf{r} \cdot \mathbf{r}_0^\dagger = r(y_0 \sin\theta - z_0 \cos\theta);\ H_0^{(1)}(x)\Big|_{x\to\infty} \approx \sqrt{\frac{2}{\pi x}} e^{i(x - \frac{\pi}{4})}, \tag{14}$$

where r is the length of $\mathbf{r}$; $\sin\theta = y/r$; $\cos\theta = z/r$. Substituting Eq.(14) and Eq.(5) into Eq.(13), we obtain:

$$G(y, z, \omega; y_0, 0) \approx i\sqrt{\frac{1}{2\pi k_0 r}} e^{i(k_0 r - \frac{\pi}{4})} e^{-ik_0 y_0 \sin\theta} \frac{\cos\theta^*}{\cos\theta^* + \beta_0(\omega)}. \tag{15}$$

After substituting Eq.(15) into Eq.(9), the extraordinary reflection becomes:

$$p_{re} \approx -\sqrt{\frac{k_0}{2\pi r}} e^{i(k_0 r - \frac{\pi}{4})} \frac{\cos\theta^*}{\cos\theta^* + \beta_0(\omega)} \int_{-\infty}^{\infty} \tilde{\beta}(y_0, \omega) p(y_0, 0, \omega) e^{-ik_0 y_0 \sin\theta_{re}} dy_0. \tag{16}$$

Further, after applying Born approximation to Eq.(16) and expanding it by Eq.(1) and Eq.(3), p_{re} becomes:

$$p_{re} \approx -\sqrt{\frac{2k_0}{\pi r}} \frac{p_{i0}(\omega) \exp[i(k_0 r - \frac{\pi}{4})] \cos\theta^* \cos\theta_i}{[\cos\theta^* + \beta_0(\omega)][\cos\theta_i + \beta_0(\omega)]} \int_{-\infty}^{\infty} \tilde{\beta}(y_0, \omega) e^{ik_0 y_0 (\sin\theta_i - \sin\theta_{re})} dy_0. \tag{17}$$

Now we consider our proposed SAI:

$$Z_n(y, \omega) = A\left[1 - i\tan\frac{\psi(y)}{2}\right]; \beta_0(\omega) = \frac{\rho_0 c_0}{2A}. \tag{18}$$

After substituting Eq.(18) into Eq.(3) and Eq.(17), we obtain the ordinary reflection and the extra reflection: extraordinary reflection:

$$p_{ro} \propto \frac{2A\cos\theta_i - \rho_0 c_0}{2A\cos\theta_i + \rho_0 c_0} \exp[ik_0(y\sin\theta_{ro} + z\cos\theta_{ro})], \tag{19}$$

$$p_{re} \propto \int_{-\infty}^{\infty} e^{i\psi(y)} e^{ik_0 y(\sin\theta_i - \sin\theta_{re})} dy. \tag{20}$$

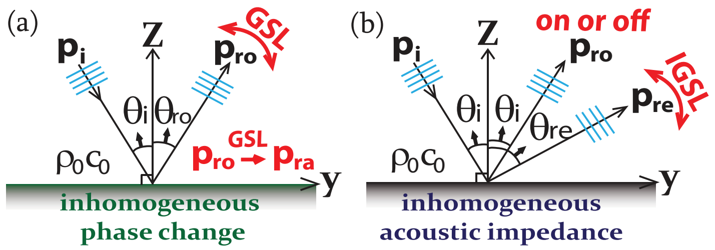

FIG. 2: (a) For a flat interface with an inhomogeneous phase change, the angle of p_{ro}, i.e., θ_{ro}, is tweaked in a fashion of GSL. The manipulated "ordinary reflection" is called to be the anomalous reflection p_{ra} in terms of GSL. [6] (b) For a flat interface with an inhomogeneous SAI, $\theta_{ro} = \theta_i$ without influence, while p_{re} occurs simultaneously and θ_{re} is controlled by IGSL, implying double reflections. If SAI is properly controlled, p_{ro} can be switched off.

Here note that in our case we are able to create *double reflections* by means of SAI inhomogeneity.

Eq.(20) is a Dirac Delta if we consider $\psi(y)$ to be a linear term as the first order approximation. Or else, we know that the integral in Eq.(20) will reach the maximum by imposing the stationary phase approximation, i.e.,

$$\sin\theta_{re} - \sin\theta_i = \frac{1}{k_0}\frac{d\psi(y)}{dy}. \tag{21}$$

Although Eq.(21) corresponds to the form of the generalized Snell's law of reflection (GSL) [6], the variables in the two situations are different. Starting from Eq.(18) and ending up with Eq.(21), we provide the insight between our designed SAI and the direction of p_{re}, without considering the phase in terms of wave propagation. We name Eq.(21) as IGSL in acoustics, as the design principle of the SAI Eq.(18).

According to Eq.(19), if $A = (\rho_0 c_0)/(2\cos\theta_i)$, we can switch off p_{ro}. Therefore Eq.(18) becomes

$$Z_n(y,\omega) = \frac{\rho_0 c_0}{2\cos\theta_i}\left[1 - i\tan\frac{\psi(y)}{2}\right]. \tag{22}$$

II. DIFFERENCES BETWEEN GSL AND IGSL

Although GSL is not our topic in this paper, the same appearance of IGSL and GSL may cause the false impression that our IGSL is the same as GSL. Actually their mechanisms are totally different.

In terms of phase inhomogeneity, the anomalous reflection p_{ra} actually corresponds to the situation when the ordinary reflection p_{ro} is steered toward a "wrong" direction governed by GSL [6], illustrated in Fig. 2(a). There is only one single direction of reflection all the while. On the contrary in terms of SAI inhomogeneity, it is found that IGSL cannot alter p_{ro} by an SAI interface, but can "turn off" p_{ro} so as to provide insight into the engineering of special wavefronts by SAI interface, illustrated in Fig. 2(b). Moreover, the extraordinary reflection p_{re} governed by IGSL is an additionally unique component in acoustics, which can be "geared" along arbitrary directions, simultaneously with vanishing p_{ro}. Therefore, our proposed IGSL opens up rich effects and unprecedented applications in the community of acoustics. Additionally, GSL can even be phenomenologically considered as one subset of IGSL, when p_{ro} is turned off. In order to stress the irrelevance between IGSL and GSL again, we list the differences: 1. GSL is initiated in electromagnetism with electric properties; IGSL is initiated in acoustics with mechanical properties. 2. GSL is derived

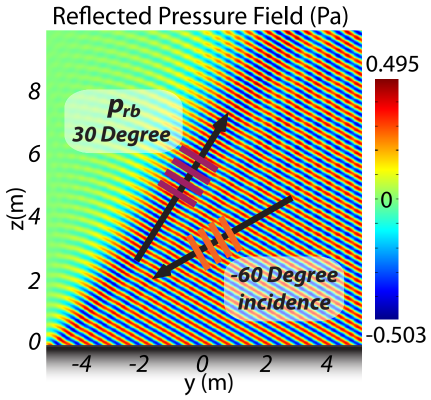

FIG. 3: The SAI Eq.(22) with $\psi(y) = (10 + 10\sqrt{3})y$ is set along the flat surface $z = 0$. In the upper space, the medium is water ($\rho_0 = 1kg/m^3$; $c_0 = 1500m/s$). An audible plane wave with unit amplitude and $\omega = 30Krad/s$ is $-60°$ obliquely incident. Only reflected acoustic pressure is plotted. The propagating path of p_{re} is noted as an arrow with purple crossbars.

from Fermat Principle, i.e., the conservation of the wave number along an interface; IGSL is derived from Green's function. The fundamental physics is distinguished. 3. The variable of GSL is phase inhomogeneity; the variable of IGSL is impedance inhomogeneity. The methods are independent. 4. GSL will only generate single reflection; IGSL not only can generate single reflection, but also can generate double reflections. 5. GSL acts upon p_{ro}; IGSL acts upon p_{re}. 6. In GSL, the anomalous reflection corresponds to the situation where p_{ro} is tweaked toward a different direction governed by GSL; in acoustics, IGSL cannot alter p_{ro} by SAI interfaces, but is capable of "turning on" or "turning off" p_{ro}.

III. SIMULATION FOR OBLIQUE INCIDENCE

In Fig. 3, we assume water ($\rho_0 = 1kg/m^3$; $c_0 = 1500m/s$ [1]) as the background medium in the upper space. The SAI Eq.(22) with the linear parameter $\psi(y) = (10 + 10\sqrt{3})y$ is set along the flat surface, and an audible ($\omega = 30Krad/s$) plane wave with a unit amplitude is obliquely incident with the incident angle $-60°$. These parameters theoretically lead to the angle of p_{re} $30°$ according to our proposed IGSL Eq.(21). Furthermore, p_{ro} vanishes thanks to the specific A chosen in Eq.(22). In Fig. 3, we find out the simulation confirms the prediction via IGSL accurately, and p_{ro} disappears as expected.

Moreover, the incident audible plane wave and p_{re} are at the same side of the normal line, confirming the possibility of the negative extraordinary reflection. The singularity due to $\tan[\psi(y)/2]$ in the imaginary part of Eq.(22) does not play a significant role because the mathematical singularity $\pm i\infty$ just occurs at singular positions, physically meaning the total reflection (reflection coefficient equals $+1$).

[1] D. T. Blackstock, *Fundamentals of physical acoustics* (Wiley, 2000).

[2] M. A. Nobile, and S. I. Hayek, Acoustic propagation over an impedance plane. J. Acoust. Soc. Am. **78**, 1325(1985).

[3] G. Taraldsen, A note on reflection of spherical waves. J. Acoust. Soc. Am. **117**, 3389 (2005).

[4] C. F. Chien, and W. W. Soroka, Sound propagation along an impedance plane. J. Sound Vib. **43**, 9 (1975).

[5] G. Taraldsen, The complex image method. Wave Motion **43**, 91 (2005).

[6] N. Yu, P. Genevet, M. A. Kats, F. Aieta, J. P. Tetienne, F. Capasso and Z. Gaburro, Light propagation with phase discontinuities: generalized laws of reflection and refraction. Science **334**, 333 (2011).
